# An audit to assess whether patients under the care of a community mental health team who are taking clozapine are having their lipid profile checked annually and are given lifestyle advice and have had a QRISK3 assessment

**DOI:** 10.1192/bjo.2021.272

**Published:** 2021-06-18

**Authors:** Elisabeth Linley-Adams, Bethan Harris

**Affiliations:** Cardiff and Vale University Healthboard

## Abstract

**Aims:**

‘All cause’ mortality is higher among patients with serious mental illness than the general population and a significant contributor from this is cardiovascular disease. Mean triglyceride levels have been shown to double and cholesterol levels to increase by at least 10% after 5 years’ treatment with clozapine. NICE guidelines state all patients should have their lipids measured at baseline, 3 months after starting treatment with a new antipsychotic, and then annually.

The first aim of our audit was to identify whether patients who had been on clozapine for at least 3 months from our community mental health team (CMHT) who were not taking cholesterol lowering medication are having their lipid profile checked annually. The second aim was to see whether these patients have high total cholesterol levels and whether they had had a documented discussion about exercise, diet or lifestyle and a QRISK3 assessment.

**Method:**

We constructed a list of 56 patients who were taking clozapine from the CMHT. We excluded 17 patients who were on cholesterol lowering medication and would have excluded any patients who had been on clozapine for less than 3 months. We then looked at whether the patients had had a lipid profile and identified patients with a cholesterol level >5.0 to indicate a ‘high cholesterol level.’ We then searched through the last year of each of the patient's case notes to see whether they had had a QRISK assessment or lifestyle advice by searching for the words ‘diet, exercise, lifestyle and QRISK’.

**Result:**

36 of the 39 (92%) patients had lipid levels checked in the last 12 months. 21 of the 39 (54%) patients had a cholesterol over 5.0. 9 of the 39 (23%) patients had a documented discussion regarding lifestyle, diet or exercise in the last year. 0 of the 39 (0%) patients had a documented QRISK3 assessment.

**Conclusion:**

Most (92%) patients from the CMHT had their lipid profile checked in the last year. 54% had total cholesterol level over 5.0. Only a small proportion (23%) had documented lifestyle discussion and none of the patients had a QRISK3 assessment. The results will be presented to the CMHT and we will organise teaching on giving lifestyle advice and QRISK3 assessments.

